# Erythrocyte superoxide dismutase as a biomarker of septic acute kidney injury

**DOI:** 10.1186/s13613-016-0198-5

**Published:** 2016-10-06

**Authors:** Nara Aline Costa, Ana Lúcia Gut, Paula Schmidt Azevedo, Suzana Erico Tanni, Natália Baraldi Cunha, Eloá Siqueira Magalhães, Graziela Biude Silva, Bertha Furlan Polegato, Leonardo Antonio Mamede Zornoff, Sergio Alberto Rupp de Paiva, André Luís Balbi, Daniela Ponce, Marcos Ferreira Minicucci

**Affiliations:** 1 Department of Internal Medicine, Botucatu Medical School, UNESP – Univ Estadual Paulista, Rubião Júnior s/n, Botucatu, SP CEP: 18618-970 Brazil; 2 Department of Food and Experimental Nutrition, Faculty of Pharmaceutical Science, University of São Paulo, São Paulo, SP Brazil

**Keywords:** Superoxide dismutase, Acute kidney injury, Sepsis, Oxidative stress

## Abstract

**Background:**

Oxidative stress is a key feature of sepsis and could be a common pathophysiological pathway between septic shock and acute kidney injury (AKI) Our objective was to evaluate the erythrocyte superoxide dismutase (SOD1) activity as predictor of AKI in patients with septic shock.

**Methods:**

This is a prospective observational study that evaluated 175 consecutive patients over the age of 18 years with septic shock upon intensive care unit (ICU) admission. However, 43 patients were excluded (27 due to AKI at ICU admission). Thus, 132 patients were enrolled in the study. At the time of the patients’ enrollment, demographic information was recorded. Blood samples were taken within the first 24 h of the patient’s admission to determine the erythrocyte SOD1 activity. All patients were followed throughout the ICU stay, and the development of AKI was evaluated. In addition, we also evaluated 17 control subjects.

**Results:**

The mean age of patients with septic shock was 63.2 ± 15.7 years, 53 % were male and the median ICU stay was 8 days (4–16). Approximately 50.7 % developed AKI during the ICU stay. The median erythrocyte SOD1 activity was 2.92 (2.19–3.92) U/mg Hb. When compared to control subjects, septic shock patients had a higher serum malondialdehyde concentration and lower erythrocyte SOD1 activity. In univariate analysis, erythrocyte SOD1 activity was lower in patients who developed AKI. The ROC curve analysis revealed that lower erythrocyte SOD1 activity was associated with AKI development (AUC 0.686; CI 95 % 0.595–0.777; *p* < 0.001) at the cutoff of <3.32 U/mg Hb. In the logistic regression models, SOD1 activity higher than 3.32 U/mg Hb was associated with protection of AKI development when adjusted by hemoglobin, phosphorus and APACHE II score (OR 0.309; CI 95 % 0.137–0.695; *p* = 0.005) and when adjusted by age, gender, chronic kidney disease, admission category (medical or surgery) and APACHE II score (OR 0.129; CI 95 % 0.033–0.508; *p* = 0.003).

**Conclusions:**

In conclusion, our data suggest that erythrocyte SOD1 activity could play a role as an early marker of septic AKI and could be seen as a new research avenue in the field of biomarker in AKI. However, our study did not show a strong correlation between SOD activity and AKI. Nevertheless, these original data do warrant further research in order to confirm or not this hypothesis.

## Background

Acute kidney injury (AKI) is one of the most serious and frequent complications of sepsis in critically ill patients [1]. It is estimated that AKI develops within the first 24 h in 51–64 % of patients with sepsis with hypotension [2]. Septic shock is the major cause of death in the intensive care unit (ICU), and the presence of AKI in these patients leads to an increase in mortality [3].

The diagnosis of AKI is usually based on either an elevation of serum creatinine or the detection of oliguria [4]. However, these tests have limited sensitivity and specificity for the detection of renal dysfunction [5]. In this way, the development of biomarkers for the early detection of AKI is a research priority [6]. Some AKI markers are currently being intensely studied in several different clinical situations, mainly in critically ill patients [7–11]. However, some of these markers could be influenced by systemic inflammation and infections, and early AKI predictors in patients with sepsis are still lacking.

A barrier to uncovering new specific biomarkers for sepsis-induced AKI is a lack of understanding of its pathophysiology. It is known that the oxidative stress caused by sepsis is one factor responsible for its development [12, 13]. Oxidative stress is regulated by a balance between the rates of reactive oxygen species (ROS) generation and antioxidant systems, including superoxide dismutase (SOD), catalase, glutathione peroxidase, thioredoxin and vitamins [14]. SOD is considered to be the first line of defense against ROS because it catalyzes the dismutation of superoxide radicals to a less reactive product, hydrogen peroxide. Increased ROS production or an impaired antioxidant system will lead to oxidative damage to proteins, lipids and DNA. There is no ideal marker for oxidative stress; however, with regard to lipid peroxidation, malondialdehyde (MDA) is one of the most commonly used markers for the determination of oxidative stress in humans [15].

Experimental and clinical studies of chronic kidney disease showed that SOD levels were reduced and markers of oxidative damage were increased in this scenario [16–18]. In addition, strategies to increase SOD activity with recombinant SOD molecules and gene transfer led to reduced inflammation and oxidative stress in experimental models of ischemia–reperfusion and contrast-induced AKI [19–21]. It is also interesting to observe that in patients submitted to kidney transplantation, MDA levels were predictors of graft dysfunction [22, 23]. Despite the importance of oxidative stress, SOD1 activity has not yet been evaluated as predictor of AKI in patients with septic shock.

Therefore, the aim of our study was to evaluate the erythrocyte SOD1 activity as predictor of AKI in patients with septic shock.

## Methods

This prospective observational study was conducted from May 2014 to June 2015 with patients admitted to the intensive care unit of our hospital. The protocol was approved by the ethics committee of our institution (30457414.7.0000.5411). Written informed consent was obtained from all patients or relatives prior to their inclusion in the study.

This study was a subanalysis of a larger unpublished study, which evaluated the relationship between oxidative stress and mortality in patients with septic shock. The sample size was calculated using the Fisher and Belle formula, with the following variables: mortality rate of septic shock 40–60 %, 95 % confidence interval and 10 % sample error. The result was a minimum sample size of 96 patients.

Patients were eligible for enrollment if they were 18 years or older and had septic shock on ICU admission. Exclusion criteria were patients with AKI at ICU admission, patients with stage 4 or 5 chronic kidney disease (CKD) (creatinine clearance lower than 30 mL/min/1.73 m^2^), a delay in septic shock diagnosis (longer than 24 h), pregnant women, patients with confirmed brain death, patients in palliative care, and those who used vasoactive drugs for <24 h.

At the time of the patients’ enrollment, demographic information, the Acute Physiology and Chronic Health Evaluation (APACHE II) score and the Sequential Organ Failure Assessment (SOFA) score were recorded. Blood samples were taken within the first 24 h of the patient’s admission, after hemodynamic stabilization, to determine the erythrocyte SOD1 activity. All patients were followed during their ICU stay, and the development of AKI was evaluated. Mortality rate and the length of the ICU stay were also recorded.

Septic shock was defined according to Survival Sepsis guidelines [24], and AKI was defined according to Kidney Disease Improving Global Outcomes (KDIGO) criteria, using the increase in serum creatinine ≥0.3 mg/dL within 48 h or increase in serum creatinine ≥1.5 times baseline within 7 days [25]. Classification of AKI remains challenging because serum creatinine measurements, before the inciting illness, are often missing. In the absence of a standard method to accommodate missing values, investigators have used a variety of surrogate measures. One of these methods is to use the baseline creatinine as the lowest creatinine value in the last 6 months before AKI or, for those without this measurement, the lowest value achieved during hospitalization in the absence of dialysis [26, 27]. This was the definition we used of baseline creatinine in our study. CKD was defined as glomerular filtration rate lower than 60 mL/min/1.73 m^2^ using baseline creatinine and CKD Epidemiology Collaboration equation (CKD-EPI) (GFR = 141 × min(Scr/*κ*, 1)*α* × max(Scr/κ, 1) − 1.209 × 0.993 Age × 1.018 [if female] _ 1.159 [if black], where Scr is serum creatinine, *κ* is 0.7 for females and 0.9 for males, *α* is −0.329 for females and −0.411 for males, min indicates the minimum of Scr/*κ* or 1, and max indicates the maximum of Scr/*κ* or 1) [28].

To understand the behavior of erythrocyte SOD1 activity in our patients with septic shock, we also evaluated these variables in 17 control subjects (non-hospitalized individuals without any acute disease).

### Laboratorial analysis

Total serum levels of sodium, potassium, phosphorus, C-reactive protein (CRP), albumin, creatinine and urea were measured using the dry chemistry method (Ortho-Clinical Diagnostics VITROS 950^®^, Johnson & Johnson), and lactate was measured using a Roche OMNI^®^ S Blood Gas Analyzer. Hemograms were performed with a Coulter STKS hematologic autoanalyzer (Luton/Bedfordshire, UK).

### Serum MDA concentration

Serum MDA levels were analyzed based on the reaction with thiobarbituric acid by high-performance liquid chromatography (HPLC) according to a method developed by Katepe [29]. UV detection was performed at 532 nm.

### Erythrocyte SOD1 activity

The SOD enzyme activity in erythrocytes was determined in a Lyasis biochemical analyzer according to methodology recommended by the manufacturer (Ransod kit; Randox Laboratories Ltd., Crumlin, Antrim, UK) [30].

### Statistical analysis

Data are expressed as the mean ± SD, the median (including the lower and upper quartiles) or percentage. Comparisons between two groups for continuous variables were made using Student’s *t* test or the Mann–Whitney *U* test. Comparisons between two groups for categorical variables were made using the *χ* ^2^ test or Fisher’s exact test. Comparisons between continuous variables were made using Spearman correlation test. Receiver operating characteristic (ROC) curve analysis was performed to determine the performance of erythrocyte SOD1 activity to predict AKI development. A logistic regression model was used to predict AKI. We constructed two regression models. Erythrocyte SOD1 activity was tested as a categorical variable (>3.32 U/mg Hb, cutoff obtained by ROC curve). In the first model, erythrocyte SOD1 activity was adjusted with parameters that exhibited significant differences in the univariate analysis. The only exceptions were variables with high collinearity among them (urea, creatinine, potassium, MDA, CKD and SOFA score). In the other, SOD1 was adjusted by age, gender, CKD, admission category (medical or surgery) and APACHE II score. Data analysis was performed using SigmaPlot software for Windows v12.0 (Systat Software Inc., San Jose, CA, USA). *p* values lower than 0.05 were considered statistically significant.

## Results

**Fig. 1:**
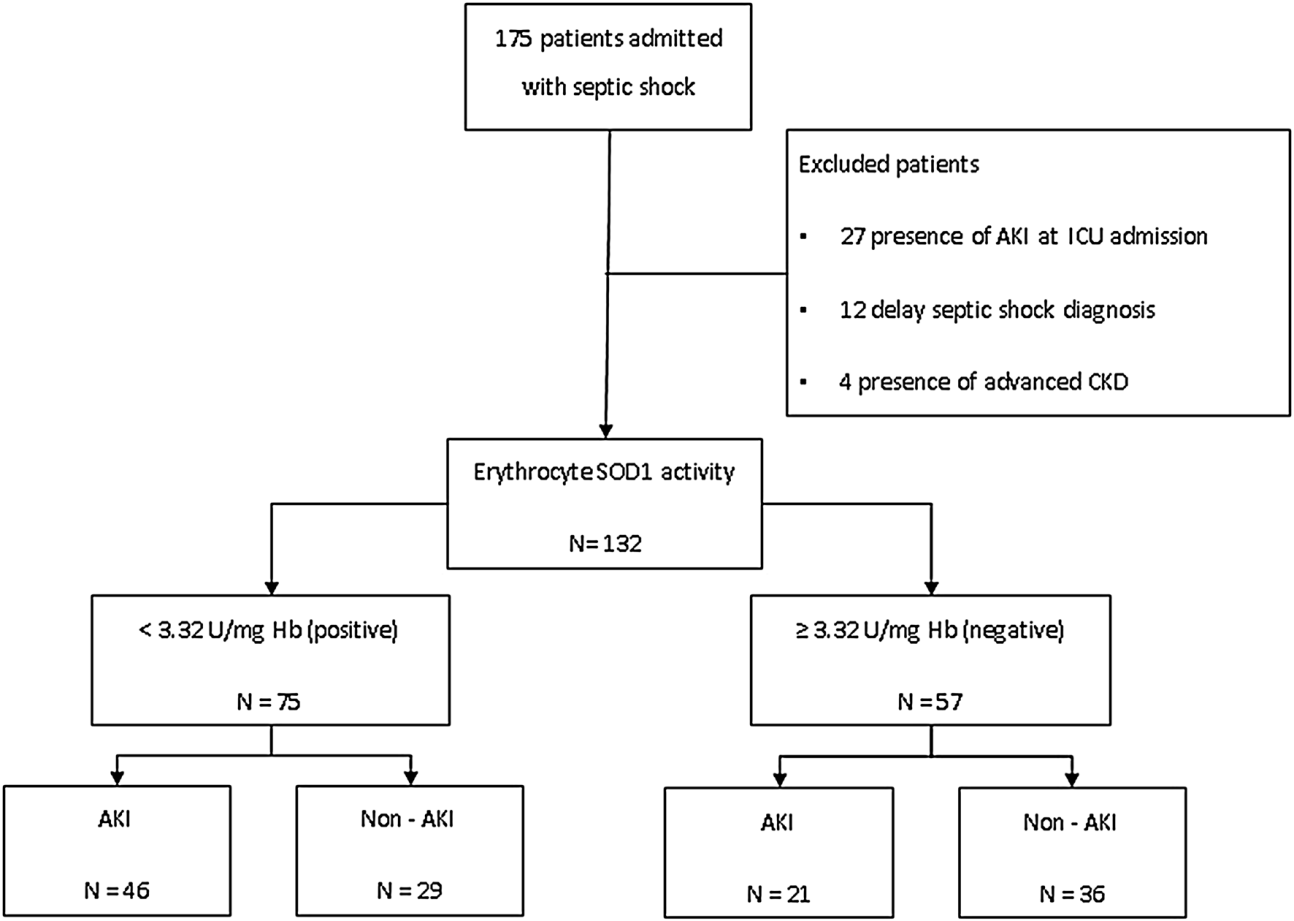
Flow diagram of studied patients with septic shock

During the study, 175 consecutive patients were admitted with a diagnosis of septic shock in the ICU; however, 43 patients were excluded (presence of AKI at ICU admission: 27 patients; delay in septic shock diagnosis: 12 patients; presence of advanced chronic kidney disease: 4 patients). Thus, we evaluated 132 patients (Fig. 1). The mean age was 63.2 ± 15.7 years, 53 % were male and the median length of ICU stay was 8 days (4–16). The mortality rate during the ICU stay was 65.9 %. Median erythrocyte SOD1 activity was 2.92 (2.19–3.92) U/mg Hb.

Among those patients with septic shock, 50.7 % developed AKI during the ICU stay. We have baseline serum creatinine levels in 37 patients of 67 patients who developed AKI, and in 30 patients, we used the lowest value achieved during hospitalization in the absence of dialysis. Regarding KDIGO stages, 17.9 % were classified as KDIGO 1, 19.4 % as KDIGO 2 and 62.7 % as KDIGO 3. The median time for AKI diagnosis based on KDIGO criteria was 2.3 days after admission. Among patients who developed AKI, 24.5 % needed dialysis during the ICU stay. The APACHE II and SOFA scores were also higher in patients in the AKI group. The mortality rate increased more than 30 % in patients with AKI. There were no differences between groups in the other demographic and clinical data (Table 1).

**Table 1:** Demographic and clinical data of 132 patients with septic shock

Variable	Acute kidney injury		*p* value
	Yes (*n* = 67)	No (*n* = 65)	
Age (years)	67.0 (59.0–75.0)	64.0 (50.5–72.5)	0.20
Male, *n* (%)	35.0 (52.2)	35 (53.8)	0.99
APACHE II score	18.7 ± 6.0	16.0 ± 6.4	<0.001
SOFA score	10.0 (8.0–12.0)	8.0 (7.0–9.5)	<0.001
RBC transfusions, *n* (%)	36 (53.7)	29 (44.6)	0.38
Steroids, *n* (%)	38 (56.7)	30 (46.2)	0.30
Admission category, *n* (%)			
Medical	18 (26.9)	27 (41.5)	0.11
Surgery	49 (73.1)	38 (58.5)	
Sepsis focus, *n* (%)			0.46
Respiratory	42 (62.7)	34 (52.3)	
Abdominal	16 (23.8)	16 (24.6)	
Urinary	3 (4.5)	4 (6.2)	
Others	6 (9.0)	11 (16.9)	
Dialysis, *n* (%)	11 (16.4)	0 (0)	0.002
CKD, *n* (%)	57 (85.1)	7 (10.8)	<0.001
MV, *n* (%)	60 (89.6)	55 (84.6)	0.56
Length of ICU stay (days)	7.0 (5.0–15.0)	9.0 (4.0–16.5)	0.63
ICU mortality, *n* (%)	52.0 (77.6)	35.0 (53.8)	0.007

Data are expressed as the mean ± SD, median (including the lower and upper quartiles) or percentage

*APACHE II* Acute Physiology and Chronic Health Evaluation, *SOFA* Sequential Organ Failure Assessment, *RBC* red blood cells, *CKD* chronic kidney disease, *MV* mechanical ventilation, *ICU* intensive care unit

The laboratory data are presented in Table 2. Patients who developed AKI had higher levels of potassium, phosphorus, MDA, urea and creatinine and lower levels of hemoglobin at baseline than patients without renal injury.

**Table 2:** Laboratory data of 132 patients with septic shock

Variable	Acute kidney injury		*p* value
	Yes (*n* = 67)	No (*n* = 65)	
Lactate (mmol/L)	2.2 (1.4–3.5)	2.1 (1.1–3.4)	0.78
Hemoglobin (g/dL)	11.0 (9.1–12.0)	11.6 (10.0–12.7)	0.025
Hematocrit (%)	32.0 ± 6.4	34.1 ± 5.7	0.06
Leukocytes (10^3^/mm^3^)	16.6 (12.2–21.6)	16.3 (12.2–24.0)	0.89
Sodium (mmol/L)	145 (140–149)	141 (137–148)	0.21
Potassium (mmol/L)	4.5 ± 0.9	4.1 ± 0.7	0.013
Phosphorus (mg/dL)	4.7 (3.4–6.6)	3.9 (2.7–4.9)	0.004
Glycemia (mg/dL)	145 (118–186)	146 (115–184)	0.87
CRP (mg/dL)	36.0 (28.0–44.1)	32.0 (8.5–41.5)	0.07
MDA (µmol/L)	1.65 (1.02–2.52)	1.25 (0.60–2.01)	0.009
Albumin (g/dL)	2.3 (2.0–2.5)	2.1 (1.8–2.8)	0.61
Urea (mg/dL)	95 (67–159)	53 (32–86)	<0.001
Creatinine (mg/dL)	2.1 (1.6–2.6)	0.7 (0.5–1.1)	<0.001

Data are expressed as median (including the lower and upper quartiles)

*CRP* C-reactive protein

**Fig. 2:**
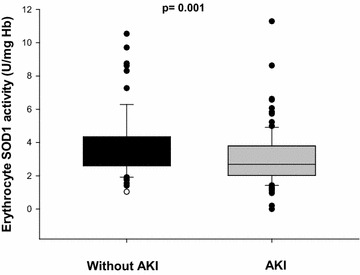
Erythrocyte SOD1 activity in patients with septic shock

**Fig. 3:**
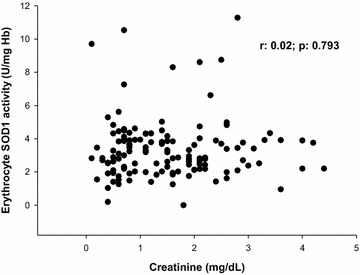
Correlation of serum creatinine at ICU admission with erythrocyte SOD1 activity

Erythrocyte SOD1 activity was lower in patients who developed AKI [non-AKI: 3.62 (2.59–4.34) vs AKI: 2.62 (2.02–3.63) U/mg Hb; *p* = 0.001; Fig. 2]. It is also important to observe that, although creatinine levels at ICU admission were higher in patients who developed AKI during ICU stay, erythrocyte SOD1 activity was not correlated with serum creatinine at ICU admission (Fig. 3). Our patients with septic shock had higher levels of serum MDA concentration [septic shock (*n*:132): 1.43 (0.77–2.19) µmol/L; controls (*n*:17): 0.86 (0.49–1.07) µmol/L; *p* = 0.003] and lower erythrocyte SOD1 activity [septic shock (*n*:132): 2.92 (2.19–3.92) U/mg Hb; controls (*n*:17): 4.35 (3.32–4.68) U/mg Hb; *p* = 0.001] when compared to control subjects.

**Fig. 4:**
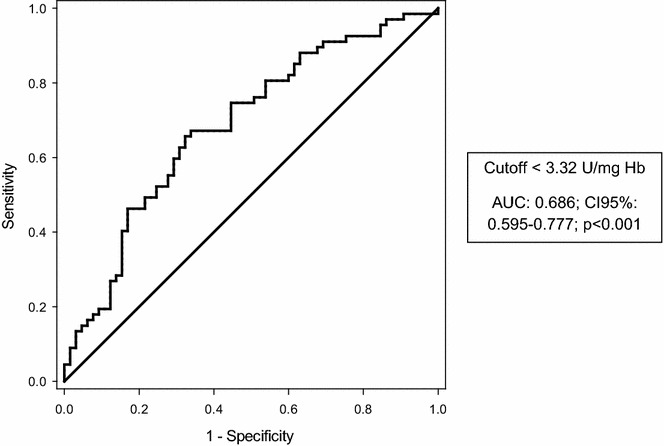
ROC curve for the association between erythrocyte SOD1 activity and AKI development

The ROC curve analysis revealed that lower erythrocyte SOD1 activity was associated with AKI development (AUC 0.686; CI 95 % 0.595–0.777; *p* < 0.001) at the cutoff of <3.32 U/mg Hb [sensibility: 68.7 % (CI 95 % 57.6–79.8 %); specificity: 55.4 % (CI 95 % 43.3–67.5 %); positive predictive value: 61.3 % (CI 95 % 50.3–72.3 %); negative predictive value: 63.2 % (CI 95 % 50.7–75.7 %)] (Table 3; Fig. 4).

**Table 3:** Cross-tabulation of erythrocyte SOD1 activity and acute kidney injury development

Erythrocyte SOD1 activity	Acute kidney injury		
	Yes	No	Total
<3.32 U/mg Hb (positive)	46	29	75
≥3.32 U/mg Hb (negative)	21	36	57
Total	67	65	132

In the logistic regression models, SOD1 activity higher than 3.32 U/mg Hb was associated with protection of AKI development when adjusted by hemoglobin, phosphorus and APACHE II score (OR 0.309; CI 95 % 0.137–0.695; *p* = 0.005) and when adjusted by age, gender, CKD, admission category (medical or surgery) and APACHE II (OR 0.129; CI 95 % 0.033–0.508; *p* = 0.003; Table 4).

**Table 4:** Logistic regression model for the prediction of acute kidney injury in 132 patients with septic shock

Variable	OR	IC 5–95 %	*p* value
SOD1 >3.32 U/mg Hb^a^	0.326	0.158–0.671	0.002
SOD1 >3.32 U/mg Hb^b^	0.129	0.033–0.508	0.003
SOD1 >3.32 U/mg Hb^c^	0.309	0.137–0.695	0.005

^a^Unadjusted

^b^Adjusted by gender, age, chronic kidney disease, admission category and APACHE II score

^c^Adjusted by APACHE II, phosphorus and hemoglobin

## Discussion

The objective of our study was to evaluate the erythrocyte SOD1 activity as marker of AKI in patients with septic shock. Although our study is a preliminary data, it showed for the first time in septic shock patients that erythrocyte SOD1 activity plays a role as a biomarker of AKI.

Sepsis is defined as the presence (probable or documented) of infection together with a systemic inflammatory response [24]. Septic shock is defined as sepsis-induced hypotension that persists despite adequate fluid resuscitation [24]. Regardless of the creation of international guidelines for the early diagnosis and treatment of septic shock, its mortality remains high. This mortality rate is even higher when septic shock is associated with AKI. In our study, the mortality rate increased more than 30 % in patients with AKI. Thus, the early detection and treatment of patients who develop AKI are extremely important.

Several studies were performed in different clinical settings to identify biomarkers for AKI prediction, most of them in critically ill patients [5–8, 10, 11]. Until now, there have been four major categories of biomarkers for AKI in these patients: functional markers, such as serum creatinine; up-regulated proteins, such as NGAL and IL-18; low molecular weight proteins, such as urine cystatin C; and enzymes, such as alpha-glutathione *s*-transferase and pi-glutathione *s*-transferase [10]. Recently, the combination of urine tissue inhibitor of metalloproteinase 2 and insulin-like growth factor binding protein 7, known as TIMP-2 × IGFBP7, was allowed for marketing by the US Food and Drug Administration [11]. Up-regulation of these markers in patients with AKI has been proposed to reflect their growth-inhibitory function due to G1 cell-cycle arrest, a known consequence of AKI [11]. Despite all of these new markers, the prediction of AKI in patients with septic shock is still challenging. A barrier to uncovering new biomarkers for this scenario is a lack of understanding of the pathophysiology of sepsis-induced AKI [12, 13]. However, oxidative stress is a key feature of sepsis and could be a common pathophysiological pathway between septic shock and AKI [31].

Oxidative stress is characterized as an imbalance between ROS and antioxidant pathways, leading to oxidative damage to proteins, lipids and DNA. At the nanomolar scale, ROS play an important role in physiological processes by functioning as a second messenger in signal transduction pathways [32]. However, at the micromolar scale, ROS is associated with oxidative injury [32]. SOD is considered the first line of defense against ROS, consisting of three isoenzymes: copper/zinc SOD (SOD1), which is localized in the cytosol, nucleus and intermembrane space of mitochondria; manganese SOD (SOD2), which occurs in the mitochondrial matrix; and extracellular SOD (SOD3) [33]. SOD1 is the most abundant member of the family of antioxidant enzymes, representing approximately 90 % of SODs [34].

Experimental studies showed that SOD concentration and activity are reduced in chronic kidney injury [17, 19–21]. Vaziri et al. showed that SOD concentration was reduced and nicotinamide adenine dinucleotide phosphate (NADPH) oxidase expression was increased in the kidneys of rats submitted to nephrectomy [17]. Additionally, in studies with recombinant SOD molecules and SOD gene transfer, the animals submitted to these interventions presented reduced inflammation and oxidative stress in experimental models of ischemia–reperfusion and contrast-induced AKI [19–21].

In a clinical study with 134 individuals, Magalhães et al. compared hemodialyzed patients with healthy controls and showed that the activity of SOD is reduced in patients with chronic renal failure undergoing hemodialysis [16]. In our study, the activity of erythrocyte SOD1 was also lower in patients who developed AKI. This association remains statistically significant, even after adjustments for confounding variables in multivariate regression analysis. It is also important to note that erythrocyte SOD1 activity was lower and serum MDA concentration was higher in septic shock patients compared to control subjects. Thus, our results suggest that patients with septic shock had an impaired SOD1 response and increased oxidative stress, and this behavior was more pronounced in patients who developed AKI. In addition, this lower SOD1 activity occurred in the first 24 h of ICU admission.

The AUC of erythrocyte SOD1 activity was only 0.686; however, comparisons of AUCs of several biomarkers for AKI prediction showed also a poor performance with a huge variation in its values [35, 36]. Thus, our data suggest that erythrocyte SOD1 activity could be used as an early predictive marker of AKI.

It is important to remember that patients with chronic kidney disease have an increased risk to develop AKI. This could explain why our patients who developed AKI had higher levels of creatinine at ICU admission. Despite that, there was no correlation between creatinine at ICU admission and erythrocyte SOD1 activity. Thus, our results were not influenced by the initial difference in kidney function.

Considering that the development of AKI in patients with sepsis is related to increased mortality, the identification of early biomarkers of renal injury is extremely important. Erythrocyte SOD1 activity was associated with AKI detection 2.3 days before the KDIGO criteria. Our results have important clinical implications because we were able to identify patients at risk of developing AKI earlier. Thus, patients at risk of AKI could be treated more aggressively, improving septic shock outcomes.

We should consider some limitations of this study. We only included patients from a single medical center. Regarding AKI definition, we only have baseline serum creatinine of 55 % of patients who developed AKI, for the others we used the lowest value achieved during hospitalization in the absence of dialysis. Moreover, AKI was defined based upon the serum creatinine levels and not urine output. In addition, due to the relatively small number of patients, analysis of SOD1 according to the stage of AKI was not performed. Timing of antibiotic dosing was also not recorded, and blood was taken within the first 24 h, which is a relatively large window. Finally, the AUC of erythrocyte SOD1 activity was only 0.686, and our study is a preliminary data with a limited internal and external validity. Despite these limitations, we strongly believe that our data contribute relevant knowledge regarding the roles of SOD as early predictor of AKI in patients with septic shock.

## Conclusions

In conclusion, our data suggest that erythrocyte SOD1 activity could play a role as an early marker of septic AKI and could be seen as a new research avenue in the field of biomarker in AKI. However, our study did not show a strong correlation between SOD activity and AKI. Nevertheless, these original data do warrant further research in order to confirm or not this hypothesis.

## Abbreviations

AKI: acute kidney injury
AKIN criteria: acute kidney injury network criteria
APACHE II score: Acute Physiology and Chronic Health Evaluation II score
CI 95 %: confidence interval 95 %
CKD: chronic kidney disease
CRP: C-reactive protein
GFR: glomerular filtration rate
HPLC: high-performance liquid chromatography
ICU: intensive care unit
MDA: malondialdehyde
NGAL: neutrophil gelatinase-associated lipocalin
OR: odds ratio
ROS: reactive oxygen species
Scr: serum creatinine
SOFA score: Sequential Organ Failure Assessment score
SOD1: superoxide dismutase 1
